# WASp Is Crucial for the Unique Architecture of the Immunological Synapse in Germinal Center B-Cells

**DOI:** 10.3389/fcell.2021.646077

**Published:** 2021-06-14

**Authors:** Yanan Li, Anshuman Bhanja, Arpita Upadhyaya, Xiaodong Zhao, Wenxia Song

**Affiliations:** ^1^Department of Rheumatology and Immunology, Children’s Hospital of Chongqing Medical University, Chongqing, China; ^2^Ministry of Education Key Laboratory of Child Development and Disorders, Chongqing Key Laboratory of Child Infection and Immunity, China International Science and Technology Cooperation Base of Child Development and Critical Disorders, National Clinical Research Center for Child Health and Disorders, Chongqing, China; ^3^Department of Cell Biology and Molecular Genetics, University of Maryland, College Park, College Park, MD, United States; ^4^Department of Physics, University of Maryland, College Park, College Park, MD, United States; ^5^Institute for Physical Science and Technology, University of Maryland, College Park, College Park, MD, United States

**Keywords:** B-lymphocytes, germinal center, actin, WASp, signal transduction, immunological synapse

## Abstract

B-cells undergo somatic hypermutation and affinity maturation in germinal centers. Somatic hypermutated germinal center B-cells (GCBs) compete to engage with and capture antigens on follicular dendritic cells. Recent studies show that when encountering membrane antigens, GCBs generate actin-rich pod-like structures with B-cell receptor (BCR) microclusters to facilitate affinity discrimination. While deficiencies in actin regulators, including the Wiskott-Aldrich syndrome protein (WASp), cause B-cell affinity maturation defects, the mechanism by which actin regulates BCR signaling in GBCs is not fully understood. Using WASp knockout (WKO) mice that express Lifeact-GFP and live-cell total internal reflection fluorescence imaging, this study examined the role of WASp-mediated branched actin polymerization in the GCB immunological synapse. After rapid spreading on antigen-coated planar lipid bilayers, GCBs formed microclusters of phosphorylated BCRs and proximal signaling molecules at the center and the outer edge of the contact zone. The centralized signaling clusters localized at actin-rich GCB membrane protrusions. WKO reduced the centralized micro-signaling clusters by decreasing the number and stability of F-actin foci supporting GCB membrane protrusions. The actin structures that support the spreading membrane also appeared less frequently and regularly in WKO than in WT GCBs, which led to reductions in both the level and rate of GCB spreading and antigen gathering. Our results reveal essential roles for WASp in the generation and maintenance of unique structures for GCB immunological synapses.

## Introduction

B-cell-mediated antibody responses provide essential immune protection against infectious diseases. Antibodies and antibody responses have been explored as immune therapies and preventatives for cancer, immune disorders, and infectious diseases. Upon encountering cognate antigen, mature B-cells in B-cell follicles of the secondary lymphoid organs are activated through the B-cell antigen receptor (BCR) to proliferate, leading to the formation of germinal centers (GCs). B-cells undergo somatic hypermutation in immunoglobulin genes of the BCR in the dark zone (DZ) of GCs. Germinal center B-cells (GCBs) with mutated BCRs then migrate to the light zone (LZ), where GCBs compete with each other to engage and capture antigen through their clonal-specific BCRs (Chan and Brink, 2012; Shlomchik and Weisel, 2012; Victora and Nussenzweig, 2012). BCR’s ability to transduce signals and capture antigens is essential for GCBs to survive and differentiate into memory B-cells that mediate rapid and robust recall antibody responses and long-lived plasma cells that maintain the levels of protective antibodies (Gitlin et al., 2014; Turner et al., 2018; Shlomchik et al., 2019).

Deficiencies of actin regulators responsible for generating branched actin structures cause concurrent immune deficiencies and autoimmune diseases in humans. These actin regulators include ARPC1B, a hematopoietic-specific ARPC1 isoform of Arp2/3 complex that nucleates branched actin (Kuijpers et al., 2017; Volpi et al., 2019), and a hematopoietic-specific member of the branched actin nucleation promoting factors, Wiskott-Aldrich syndrome protein (WASp), which activates Arp2/3 (Padrick and Rosen, 2010). The immune disorders caused by deficiencies of these two actin regulators share some common characteristics, including microthrombocytopenia, immunodeficiency, eczema, and increased risk of malignancies and autoimmune manifestations (Notarangelo et al., 2008; Kuijpers et al., 2017; Volpi et al., 2019). While the impact of ARPC1B deficiency on B-cells is unknown, and the role of WASp in B-cell mediated antibody responses has been studied. Both WASp-deficient patients and mouse models, including germline and B-cell specific knockout, exhibit defects in mounting antibody responses to infections and immunizations. However, their B-cell development and B-cell differentiation into GCBs, memory B-cells, and plasmablasts are relatively normal (Becker-Herman et al., 2011; Recher et al., 2012; Simon et al., 2014). Moreover, both WAS patients and mice develop a wide range of autoreactive IgM and IgG as well as germinal centers in the absence of antigenic challenges. In chimeric mouse models with wild type (WT) T-cells, both WASp-deficient transitional B-cells and GCBs show hyper-responsiveness to BCR cross-linking and enrichment of self-reactive populations (Becker-Herman et al., 2011; Kolhatkar et al., 2015). These indicate a regulatory role of WASp-mediated branched actin in GCBs, where high-affinity antigen-specific B-cells are selected and autoreactive B-cells are eliminated.

The activation of B-cells from all subsets is initiated by the binding of the BCR to antigens. In GCs, B-cells encounter antigens on the surface of follicular dendritic cells or soluble antigen diffusing into GCs (Batista and Harwood, 2009; Cyster, 2010). Binding multivalent antigens triggers BCR aggregation in lipid rafts (Cheng et al., 1999; Sohn et al., 2008), and membrane antigens induce the formation of immunological synapses (ISs) at the contact zone between GCBs and antigen-presenting membranes. Src kinases, which constitutively reside in lipid rafts, phosphorylate the tyrosines of immunoreceptor tyrosine-based activation motifs (ITAMs) in the cytoplasmic domains of CD79a and/or CD79b, the signaling component of the BCR (Reth, 1992). The phosphorylated ITAM provides a docking site for the SH2 domain of the tyrosine kinase Syk. ITAM binding leads to Syk phosphorylation and activation. Syk then activates multiple downstream signaling pathways, including phosphatidylinositol-3 kinase, phospholipase Cγ2, and Bruton’s tyrosine kinase (Dal Porto et al., 2004; Kwak et al., 2019). Following signaling activation, several phosphatases, including SH2-containing tyrosine phosphatase 1 (SHP1) and SH2-containing phosphatidylinositol-5 phosphatase 1 (SHIP1), are recruited to ISs, leading to signal attenuation (Brauweiler et al., 2000; Franks and Cambier, 2018).

B-cell affinity maturation in GCs is antigen-driven, indicating the crucial role of BCR-antigen engagement and subsequent signaling (Mesin et al., 2016; Cirelli and Crotty, 2017; Shlomchik et al., 2019). Compared to naïve B-cells with no previous antigen experience, BCR signaling in GCBs is relatively attenuated, associated with increased activation of the phosphatases SHP and SHIP (Khalil et al., 2012; Shlomchik et al., 2019). Such attenuated BCR signaling likely increases the GCB activation threshold and their ability to distinguish between high- and low-affinity antigens (Kwak et al., 2018). Signaling activation promotes BCR internalization and intracellular transport of antigens for processing and presentation to follicular T-helper cells (Liu et al., 2013b; Avalos and Ploegh, 2014; Hoogeboom and Tolar, 2016). B-cells can capture both soluble antigens and antigens associated with APCs. Internalization of membrane-associated antigens requires higher-affinity BCRs than soluble antigens, enhancing the B-cell affinity discrimination of antigens (Batista and Neuberger, 2000).

The role of the actin cytoskeleton in BCR signaling of naïve B-cells has been extensively studied. Upon encountering cognate antigens with multi-valency or those associated with membranes, the earliest signaling triggers transient actin depolymerization, releasing surface BCRs from restraints to lateral mobility imposed by the cortical actin network (Treanor et al., 2010; Freeman et al., 2011). Following the initial depolymerization, actin polymerizes rapidly, driving BCR clustering—the formation, growth, and merging of BCR microclusters (Harwood and Batista, 2011; Song et al., 2013; Tolar, 2017). When interacting with membrane-associated antigens, actin polymerization mediates B-cell spreading, expanding the contact zone between B-cells and antigen-presenting surfaces and driving surface BCRs to the contact zone, which amplifies BCR signaling. Following spreading, B-cells undergo actin-dependent contraction (Fleire et al., 2006). In addition to facilitating the gathering of antigen-engaged BCRs to form ISs, this contraction promotes BCR signaling attenuation (Liu et al., 2013a; Seeley-Fallen et al., 2014). Branched actin polymerization, mediated by WASp and its ubiquitous homolog neuronal (N)-WASp, is essential for B-cell spreading and signaling amplification. However, N-WASp but not WASp facilitates B-cell contraction and signaling attenuation. B-cell-specific N-WASp deletion in mice also induces GCs without immunization and autoantibody production (Liu et al., 2013a). Therefore, WASp and N-WASp activated branched actin polymerization is involved in both BCR signaling amplification and attenuation in naive B-cells.

While deficiencies in actin regulators significantly impact GCBs, the exact role of the actin cytoskeleton in GCB BCR signaling has not been fully understood. Recent studies have shown a unique architecture of the GCB IS formed on antigen-presenting membranes. Distinct from the relatively smooth membrane contact of naive B-cells, GCBs generate actin- and ezrin-rich pod-like structures to contact antigen-presenting surfaces, which leads to the formation of less centralized BCR microclusters (Nowosad et al., 2016; Kwak et al., 2018). The stability of this specialized IS depends on BCR antigen-binding affinity, consequently enhancing GCBs’ ability to distinguish antigen affinity (Kwak et al., 2018). The unique pod-like structure of GCB ISs and the particular impact of deficiencies in actin regulators for branched actin on GCs suggest a distinct role of the actin cytoskeleton in GCB ISs.

This study examined the mechanism by which WASp promoted polymerization of branched actin in the formation of GCB ISs, using WASp knockout mice expressing Lifeact-GFP that binds to F-actin and total internal reflection fluorescence microscopy. Here, we show that when interacting with planar lipid bilayers coated with a high density of Fab’ fragment of anti-BCR antibody, WT GCBs form centralized BCR signaling microclusters at plasma membrane protrusions. These centralized signaling microclusters are surrounded by regions of low signaling, in contact with the antigen-presenting membrane. Branched actin generated by WASp is required for the formation and stabilization of the unique membrane structure of the GCB ISs by generating and sustaining actin networks that support plasma membrane protrusions as well as interactions with antigen-presenting membranes in the periphery of the IS.

## Materials and Methods

### Animals

Wild type (WT, C57BL/6) and WASp knockout (WKO) mice on a C57BL/6 background were purchased from Jackson Laboratories (Cat# 000664 and 019458). A lack of WASp expression in WKO mice was verified using western blotting (Supplementary Figure 1). Lifeact-GFP mice on a C57BL/6 background were kindly provided by Dr. Roberto Weigert’s laboratory in National Cancer Institute, Maryland, United States. WKO mice expressing Lifeact-GFP were generated by crossing WKO and Lifeact-GFP mice. All work involving animals was approved by the Institutional Animal Care and Use Committee at the University of Maryland.

### Germinal Center B-Cells (GCBs)

WT and WKO mice with or without expressing Lifeact-GFP were immunized intraperitoneally with sheep red blood cells (SRBC) (Innovative Research Cat# ISHRBC10P) twice 7 days apart and euthanized at 7 days following the second immunization. Splenocytes were released from the spleens using frosted glass slides and filtered through 40 μm cell strainer (Thermo Fisher, Cat# 22-363-547). Red blood cells were lysed using ACK lysing buffer (Gibico Cat# 10492-01). GCBs were enriched using a negative selection method based on a published protocol (Cato et al., 2011). Briefly, splenocytes were incubated with biotinylated anti-CD43 (eBioscience Cat# 13-0431-82), anti-CD11c (eBioscience Cat# 13-0114-81), and anti-IgD (Southern Biotech Cat# 112008) antibodies. After washing, cells were incubated with anti-biotin microbeads (Miltenyi Biotec Cat# 130-090-485) and went through a LS column (Miltenyi Biotec Cat# 130-042-401) according to the manufacture’s recommended protocol. Cells eluted from LS columns were collected as enriched GCBs.

### Western Blotting

Western blotting was used to verify WASp-deficiency in WKO mice. Splenocytes from WT and WKO mice were lysed, and cell lysates were analyzed by western blotting, probing for WASp (Santa Cruz Cat# 365859). The blots were stripped and probed for GAPDH (Proteintech Cat# HRP-60004) as loading controls.

### Flow Cytometry

To determine the purity of enriched GCBs and compare the sizes and the surface BCR levels of GCBs from WT and WKO mice, splenocytes and isolated GCBs were stained with antibodies specific to B220 (Biolegend Cat# 103236), GL7 (Invitrogen Cat# 12-5902-82), CD95 (BD Biosciences Cat# 557653), and IgG (Biolegend Cat# 405315) and analyzed by a BD FACS Canto II flow cytometer (BD Sciences) and Flowjo software.

### Planar Lipid Bilayers (PLBs)

Mono-biotinylated Fab’ (mB-Fab’ or Fab’) was generated from the F(ab’)_2_ fragment of anti-mouse IgM + G antibody (Jackson ImmunoResearch Cat# 115-006-068) using a previously published protocol (Peluso et al., 2003). mB-Fab’ was conjugated with Alex Fluor (FA) 546 using a labeling kit (Thermo Fisher Cat# A10237) according to the manufacturer’s protocol. The PLB was prepared using liposomes made by sonicating 1,2-dioleoyl-sn-Glycero-3-phosphocholine and 1,2-dioleoyl-sn-Glycero-3-phosphoethanolamine-cap-biotin (Avanti Polar Lipids Cat# 850375 and 870273) in a 100:1 molar ratio in PBS (Sohn et al., 2008). Coverslip chambers (Thermo Fisher Cat# 155411) were incubated with the liposomes before coating with 1 μg/ml streptavidin (Jackson ImmunoResearch Cat# 016-000-084) and followed by 2 μg/ml AF546-mB-Fab’ mixed with 8 μg/ml mB-Fab’ (Fab’-PLB). For a non-stimulation control, biotinylated holo-transferrin (TF; 16 μg/ml, Jackson ImmunoResearch Cat# 015-060-050) binds Tf receptors on GCBs but does not activate the BCR was used to substitute the mB-Fab’ (Tf-PLB). To analyze surface BCRs on GCBs interacting with Tf-PLB, surface BCRs were labeled with non-biotinylated AF546-Fab’ on ice before incubating with TF-PLBs.

### Total Internal Reflection Fluorescence Microscopy (TIRF) and Image Analysis

Images were acquired using a TIRF microscope (NIKON Eclipse Ti-E TIRF, 63 × 1.49NA oil objective). Interference reflection images (IRM) and AF488 and AF546 images were acquired sequentially. To identify light-zone and dark-zone GCBs, enriched GCBs were stained with AF488-anti-CD86 (Invitrogen Cat# 53-0869-42) and PE-anti-CXCR4 (Invitrogen Cat# 12-9991-82) and then incubated with Fab’-PLBs for 5 min at 37°C before fixation with 4% paraformaldehyde. Cells with high levels of CD86 staining and low levels of CXCR4 staining were identified as light-zone GCB. After initial characterization, enriched GCBs were pre-stained with anti-CXCR4 antibodies before incubating with Fab’-PLB for all the experiments, and cells with no or low level of CXCR4 staining were identified as light-zone GCBs.

To analyze signaling and F-actin in GCBs, enriched GCBs were incubated with Fab’-PLBs or Tf-PLBs at 37°C for varying lengths of time, fixed with 4% paraformaldehyde, permeabilized with 0.05% saponin, and stained with antibodies specific for phospho-CD79a Y182 (Cell Signaling Technology Cat# 14732), phospho-SHIP1 Y1020 (Cell Signaling Technology Cat# 3941), phospho-SHP1 Y536 (Abcam Cat# ab51171), phospho-Syk (Y525/Y526) (Cell Signaling Technology Cat# 2710), phospho-Akt (S473) (Cell Signaling Technology Cat# 4060), or AF488-phalloidin (Cytoskeleton Cat# PHDG1). Cell contact area, the total (TFI) and mean fluorescence intensity (MFI) in the cell contact zone, and fluorescence intensity (FI) along lines across cells were determined based on IRM and TIRF images using NIH ImageJ.

For live-cell imaging, GCBs expressing Lifeact-GFP were incubated with Fab’-PLBs at 37°C, 5% CO_2_, and imaged by TIRF, acquiring one frame every 2 s. Cell contact area, the TFI, and the MFI in the cell contact zone were determined based on IRM and TIRF images analyzed with custom-made codes using MATLAB software (The MathWorks, Inc., Natick, MA, United States). Increasing rates of the cell contact area and the MFI of AF546-Fab’ or Lifeact-GFP in the contact zone were determined using the slopes of the contact area or MFI vs. time plots and linear regression. Kymographs of time-lapse images were generated using NIH ImageJ.

For the analysis of BCR-Fab’ cluster growth, one randomly selected kymograph was generated for each cell from time-lapse images using NIH ImageJ. AF546-Fab’ clusters, which were visually distinguishable and trackable for at least 7 min, were identified by visual inspection. Five such AF546-Fab’ clusters were randomly selected from each kymograph. The FI along each cluster track in kymographs was measured.

Phalloidin and Lifeact-GFP foci in individual cells were identified visually and manually, aided by intensity maps generated using NIH ImageJ. The MFI of all identifiable foci and the MFI of the entire contact zone of individual cells were determined using NIH ImageJ manually. F-actin foci were identified when the MFI of phalloidin foci was 2-folds or the MFI of Lifeact-GFP foci was 1.5-fold of their MFI in the cell contact zone. The relative lifetime of actin foci was determined by the duration that individual actin foci can be detected in a kymograph. The width ratio of the adherent regions, the wider side relative to the narrower side, in individual cells was determined using a randomly selected kymograph per cell generated using time-lapse IRM images and an average of four time points: 3, 5, 7, and 10 min.

### Statistical Analysis

Statistical significance was assessed using unpaired, two-tailed Student’s *t*-tests (Prism-GraphPad software) when only two groups were compared, and one-way ANOVA when 3 or more groups were compared. All data were presented as the mean ± SD (standard deviation).

## Results

### WASp-Deficiency Alters the Architecture of Germinal Center B-Cell Immunological Synapses

**FIGURE 1:**
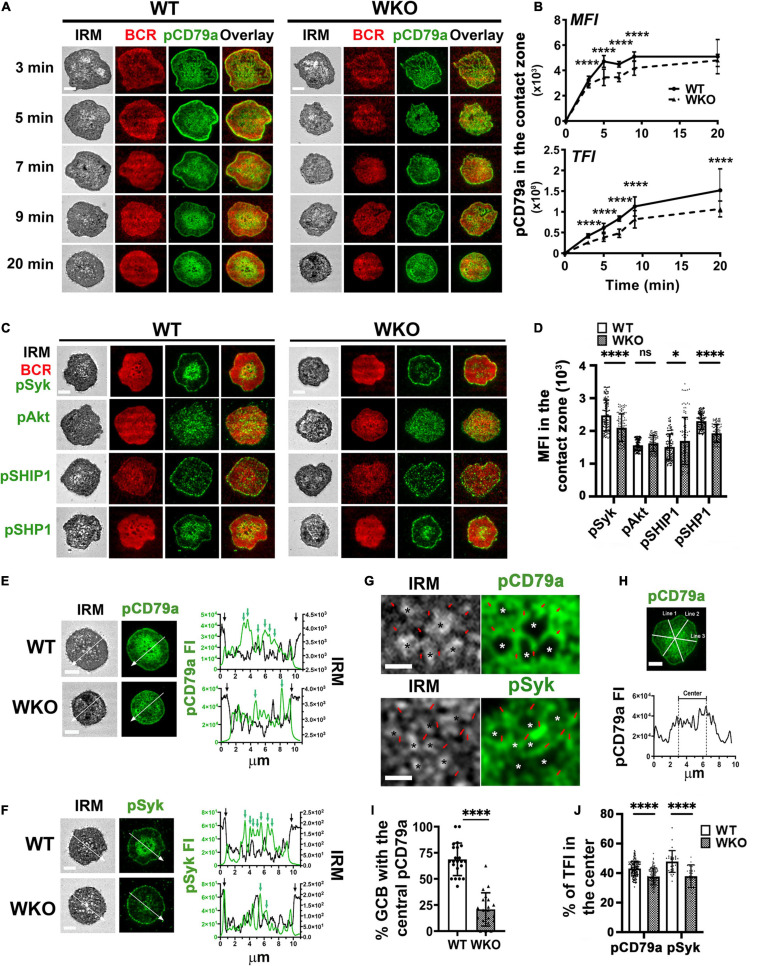
WASp knockout (WKO) reduces BCR signaling and distorts the immunological synapse (IS) of light-zone germinal center B-cells (LZ GCBs). GCBs isolated from sheep red blood cells (SRBC)-immunized wild type (WT) and WKO mice were incubated with Alexa Fluor (AF) 546-conjugated monobiotinylated Fab’-anti-mouse IgM + G tethered planar lipid bilayers (Fab’-PLB) at 37°C and fixed at different times, permeabilized, stained for phosphorylated CD79a (pCD79a), Syk (pSyk), Akt (pAkt), SHIP1 (pSHIP1), or SHP1 (pSHP1). The GCB plasma membrane contacting Fab’-PLB (contact zone) was imaged by an interference reflection microscope (IRM) and a total internal reflection fluorescence microscope (TIRF). **(A)** Representative IRM and TIRF images of WT and WKO GCBs stained for pCD79a at 3, 5, 7, 9, and 20 min. **(B)** The mean fluorescence intensity (MFI) and the total fluorescence intensity (TFI) of the pCD79a in the contact zone at different times were quantified by NIH ImageJ. The results were the average (± SD) of > 60 individual cells per condition from six independent experiments. **(C)** Representative IRM and TIRF images of WT and WKO GCBs stained for pSyk, pAkt, pSHIP1, or pSHP1 at 7 min. **(D)** The MFI of pSyk, pAkt, pSHIP1, and pSHP1 in the contact zone at 7 min was measured using NIH ImageJ. Data points represent individual cells. **(E,F)** Representative IRM and TIRF images of WT and WKO stained for pCD79a **(E)** and pSyk **(F)** at 20 min and the FI and the IRM density along the lines. In the line profiles, black arrows indicate the outer edge of the contact zone. Green arrows indicate pCD79a or pSyk FI peaks. **(G)** Enlarged representative IRM and TIRF images of the central regions in the WT GCBs stained for pCD79a and pSyk shown in **(E,F)**. Stars, plasma membrane areas that pulled away from Fab’-PLB. Red arrows, plasma membrane areas that were in close contact with Fab’-PLB. **(H)** GCBs with centralized pCD79a staining in the contact zone were identified using three randomly selected FI line profiles per cell. **(I)** Percentages of GCBs with the centralized pCD79a staining. Data points represent individual images at 5 min. **(J)** The percentages of pCD79a FI and pSyk FI in the central region indicated in **(H)**. Data points represent individual cells. Scale bars, 3 μm. The results were the average (± SD) of > 60 individual cells per condition. *n* = 3∼4. *p < 0.05 and ****p < 0.0001, unpaired Student’s *t*-test or one-way ANOVA.

To examine germinal center B-cell (GCB) immunological synapses (ISs), we isolated GCBs from sheep red blood cell (SRBC)-immunized mice (Supplementary Figure 2). GCBs were incubated with planar lipid bilayers (PLBs) coated with Alexa Fluor 546-conjugated and unconjugated monobiotinylated Fab’ fragment of anti-mouse IgM + G antibody (Fab’-PLB) or biotinylated transferrin (Tf-PLB) through biotin–streptavidin interactions (Liu et al., 2011). The Fab’-PLB elicits a maximal level of B-cell receptor (BCR) activation in naïve B-cells (Liu et al., 2011, 2012, 2013a,b; Seeley-Fallen et al., 2014). Cells were fixed at different times after the incubation and stained for phosphorylated CD79a (pCD79a), indicating activated BCRs, phosphorylated Syk (pSyk), and Akt (pAkt) as activated proximal signaling molecules, and phosphorylated SHIP1 (pSHIP1) and SHP1 (pSHP1) as activated inhibitory signaling molecules. We image the GCB plasma membrane regions that contact the PLB (contact zone) using interference reflection microscopy (IRM) and total internal reflection fluorescence microscopy (TIRF) as surface BCRs in the contact zone can directly engage Fab’-PLB. We focused on the light zone (LZ) GCBs, as the LZ is where most GCBs encounter antigen on follicular dendritic cells. We distinguished LZ GCBs among enriched GCB based on their relatively low levels of CXCR4 staining (Supplementary Figure 3; Allen et al., 2004). Phosphorylated CD79a was detected in the contact zone of both wild type (WT) and WASp knockout (WKO) LZ GCBs (Figure 1A) but not in the contact zone of GCBs interacting with Tf-PLB (Supplementary Figure 4). Both the mean fluorescence intensity (MFI) and the total fluorescence intensity (TFI) of pCD79a in the contact zone of individual LZ GCBs increased over time, peaked after ∼5 and ∼9 min incubation, respectively, and persisted at least for 20 min (Figures 1A,B). Compared to WT GCBs, the pCD79a MFI and TFI were significantly reduced in WKO GCBs (Figures 1A,B). The MFI and TFI of pSyk, pAkt, pSHIP1, and pSHP1 all increased in the contact zone of LZ GCBs over time (Supplementary Figure 5). The MFI and TFI of pSyk increased similarly as pCD79a, while the MFI and TFI of pAkt, pSHIP1, and pSHP1 peaked at 7, 3, and 3 min, respectively, and decreased afterward (Supplementary Figure 5). Compared to WT GCBs, WKO GCBs had significantly decreased levels of pSyk and pSHP1 but increased levels of pSHIP1 with no change in the level of pAkt in the contact zone (Figures 1C,D and Supplementary Figure 5).

In the contact zone of WT LZ GCBs, pCD79a and pSyk were primarily detected as numerous puncta in the central area (Figures 1E,F, upper panels, green arrows in the FI line profile) and the outer edge of the contact zone (Figures 1E,F, upper panels, black arrows in the FI line profile). The central pCD79a- and pSyk-rich area was surrounded by a pCD79a- and pSyk-poor region (Figures 1A,C,E,F). Staining of pSHP1 in the contact zone exhibited similar patterns as pCD79a and pSyk, but pAkt appeared as scattered puncta, and pSHIP1 was primarily located at the outer edge of the contact zone (Figure 1C). WKO disrupted the centralized organization of pCD79a (Figure 1E) and pSyk (Figure 1F) but did not affect the organization of the inhibitory signaling molecules pSHIP1 and pSHP1 (Figure 1C). IRM images and their intensity line profiles exhibited variability in the central part of the contact area (Figures 1E–G, left panels). We have previously shown a monotonic relationship between IRM intensity and membrane height (Lam Hui et al., 2012). The dark IRM intensities represent close apposition of the cell membrane to Fab’-PLB within the TIRF detection field, while the light IRM intensities represent the cell membrane apposition away from Fab’-PLB and the TIRF defection field. Thus, the IRM intensity variation indicates protrusions of the GCB plasma membrane in contact with the PLB (Figures 1E–G, left panels). The overlay of IRM intensity and pCD79a or pSyk FI line profiles showed that signaling puncta were located at the central protrusions that closely contacted Fab’-PLB, but much less in the membrane area surrounding the central protrusions even though it also interacted with F-ab’-PLB (Figures 1E,F, green arrows and Figure 1G, red arrows). While WKO GCBs also formed similar membrane protrusions in the contact zone, they were not centralized and less organized and associated with fewer signaling puncta (Figures 1E,F). We quantified this unique signaling organization of the GCB ISs by determining the percentage of LZ GCBs with central enrichment of pCD79a (Figure 1I) and the percentage of pCD79a or pSyk FI in the central area compared to the entire contact zone in individual cells (Figure 1J), using three randomly selected FI line profiles per cell (Figure 1H). Nearly 70% of WT LZ GCBs had such a central pCD79a organization compared to ∼20% of WKO LZ GCBs (Figure 1I). There was a significantly higher percentage of pCD79a and pSyk FI in the central area of the WT GCB contact zone compared to that in WKO GCBs (Figure 1J). Taken together, these data suggest that WASp is critical for the unique architecture of GCB ISs.

### WASp Promotes GCB Spreading on Antigen-Presenting Surfaces

**FIGURE 2:**
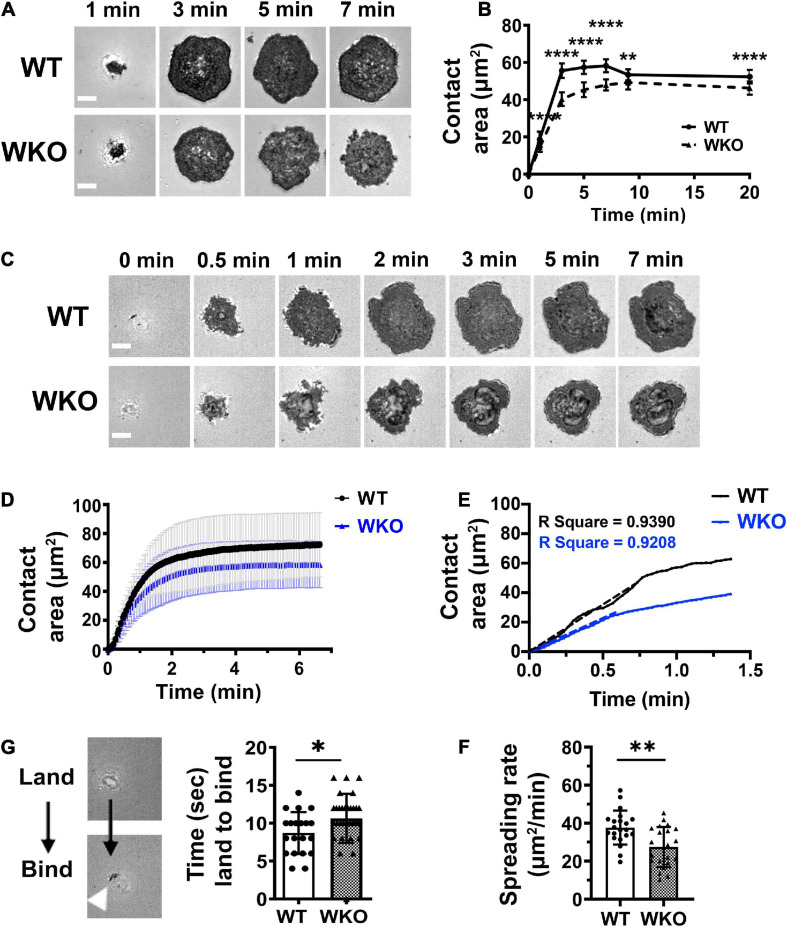
WASp contributes to GCB spreading on Fab’-PLB. GCBs isolated from SRBC-immunized mice were incubated with Fab’-PLB and either imaged after fixation **(A,B)** or live **(C–F)** by IRM. **(A)** Representative IRM images of WT and WKO GCBs fixed at indicated times. **(B)** Contact areas of fixed GCBs on Fab’-PLB over time were measured using NIH-ImageJ. The results were the average (± SD) of > 100 individual cells per condition from 7 independent experiments. **(C)** Representative time-lapse IRM images of WT and WKO GCBs. **(D)** Contact areas of WT and WKO GCBs over time using time-lapse IRM images and NIH ImageJ. The results were the average (± SD) of 20∼30 individual cells per condition from four independent experiments. **(E)** Initial spreading rates of individual GCBs were determined using the slopes of their contact area vs. time curves by linear regression. Shown is an example. **(F)** Spreading rates of WT and WKO GCBs on Fab’-PLB. **(G)** Representative IRM images (left panels) of a cell that just landed on (white shadow) or started binding (dark spots) to Fab’-PLB. The time between cell landing on and binding to Fab’-PLB (right). Data points represent individual cells. Shown are the averages (± SD) of 20∼30 individual cells for each condition. *n* = 4. Scale bars, 3 μm. *p < 0.05, **p < 0.01, and ****p < 0.0001, unpaired Student’s *t*-test or one-way ANOVA.

B-cells undergo actin-driven spreading when interacting with antigen-presenting surfaces, increasing the contact area (Fleire et al., 2006). We utilized IRM to examine the role of WASp in GCB spreading. LZ GCBs were incubated with Fab’-PLB and then either imaged after fixing at different times or imaged live by IRM. IRM images of fixed cells showed that GCBs started to bind Fab’-PLB within 1 min, rapidly spread, and reached the maximal contact area at 3 min (Figures 2A,B). Live-cell imaging was initiated when GCBs landed on the Fab’-PLB, and it took GCBs less than 2 min to reach maximal spreading (Figures 2C,D and Supplementary Video 1). WKO GCBs spread significantly less than WT GCBs (Figures 2A–D and Supplementary Video 1). In contrast to the contraction of naïve B-cells following spreading (Fleire et al., 2006), both WT and WKO GCBs maintained the maximal contact area for at least 20 min (Figures 2A–D). To analyze GCB spreading kinetics, we measured the initial GCB spreading rate using the slopes of the contact area vs. time plots and linear regression (Figure 2E). We found that WKO GCBs spread significantly slower than WT GCBs (Figures 2E,F), in addition to showing a reduction in the maximal contact area. Flow cytometry analysis showed that WT and WKO GCBs had similar forward scattering (Supplementary Figure 6), indicating that the reduced spreading rate and area of WKO GCBs were not caused by a reduction in the sizes of WKO GCBs. We also noticed that WKO GCBs took a few seconds longer than WT GCBs to transition from landing to binding to Fab’-PLB (Figure 2G). The times for cell landing and binding were determined using time-lapse images of IRM. IRM detects a shadow when a cell lands on Fab’-PLB but is not close enough to bind, and when the cell binds to Fab’-PLB, the shadow in IRM images became dark. These data suggest that WASp is required for optimal kinetics and extent of GCB spreading.

### WASp Facilitates Antigen Gathering and BCR-Antigen Microcluster Growth

**FIGURE 3:**
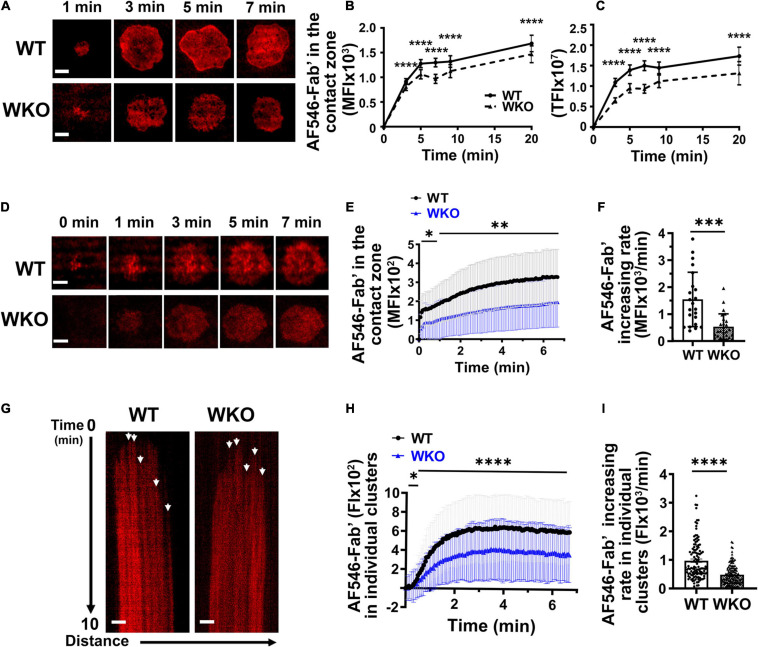
WASp promotes BCR clustering in response to Fab’-PLB. GCBs isolated from SRBC-immunized WT and WKO mice were incubated with AF546-Fab’-PLB and were imaged either after fixation at varying times or live using TIRF. **(A)** Representative AF546-Fab’ images of GCBs fixed at 1, 3, 5, and 7 min. **(B,C)** The MFI **(B)** and TFI **(C)** of AF546-Fab’ in the contact zone of fixed GCBs over time using NIH ImageJ. **(D)** Representative time-lapse AF546-Fab’ images of WT and WKO GCBs. **(E)** MFI of AF546-Fab’ in the GCB contact zone over time. **(F)** Increasing rates of AF546-Fab’ MFI in the contact zone of individual GCBs were determined using slops of AF546-Fab’ vs. time plots and linear regression, as shown in Figure 2E. Data points represent individual cells. **(G)** Representative kymographs generated using AF546-Fab’ time-lapse images of the WT and WKO GCB contact zone using NIH ImageJ. White arrows, the starts of individual AF546-Fab’ clusters. **(H)** The FI of AF546-Fab’ in individual clusters over time using kymographs and NIH ImageJ. **(I)** Increasing rates of AF546-Fab’ FI in individual clusters were analyzed as described in Figure 2E. Data points represent individual clusters. Scale bars, 3 μm. The results were the averages (± SD) of 22∼100 individual cells **(B,C,E)**, or > 120 BCR clusters and 5 clusters per kymograph per cell **(H)** for each condition. *n* = 4∼7. **p < 0.01, ***p < 0.001, and ****p < 0.0001, unpaired Student’s *t*-test or one-way ANOVA.

Upon interacting with antigen on presenting surfaces, antigen-engaged BCRs aggregate into microclusters, leading to signaling activation (Depoil et al., 2009; Tolar et al., 2009). To examine the role of WASp during this process in GCBs, we determined the relative amount of AF546-Fab’ gathered in the contact zone of individual cells and in individual microclusters, as well as their rates of increase. As AF546-Fab’ tethered on PLB clusters only when engaging surface BCRs (Supplementary Figure 4; Liu et al., 2012), AF546-Fab’ clustering reflects surface BCR clustering. GCBs were incubated with Fab’-PLB and either imaged after fixation at different times or imaged live by TIRF. The MFI and TFI of AF546-Fab’ of fixed WT and WKO GCBs peaked at 3∼5 min (Figures 3A–C), later than the time (< 3 min) when GCBs reached the maximal spreading (Figure 2B). WKO significantly reduced the MFI and TFI of AF546-Fab’ in the GCB contact zone (Figures 3A–C). Live-cell imaging confirmed the reduction of AF-546-Fab’ MFI in the WKO GCB contact zone (Figures 3D,E and Supplementary Video 2). Kinetic analysis of time-lapse images showed a drastic reduction in the rate of increase of AF546-Fab’ MFI in the WKO GCB contact zone, compared to WT GCBs (Figures 3D–F). We used kymographs from individual cells to analyze trackable AF546-Fab’ microclusters and measured their FI over time (Figure 3G, arrows). The FI of individual AF546-Fab’ microclusters in both WT and WKO GCBs peaked in less than 2 min and appeared to saturate as the intensity levels were mostly unchanged, at least for the next 5 min (Figure 3H). Furthermore, most Fab’-BCR microclusters barely moved and could therefore be tracked for several minutes in individual kymographs (Figure 3G), suggesting that they are relatively immobile. The FI and rate of increase of FI in individual AF546-Fab’ microclusters in WKO GCBs were significantly lower than those in WT GCBs (Figures 3G–I). To determine if the reductions observed in WKO GCBs were due to a decrease in surface levels of the BCR, we stained splenocytes from immunized mice for BCR (IgG), B-cell (B220), and GCB (GL7 and CD95) markers. Flow cytometry analysis showed that the IgG level on WKO GCBs was not reduced but rather slightly increased, compared to WT GCBs (Supplementary Figure 7). Thus, our results suggest that WASp facilitates antigen gathering by promoting the growth of antigen-engaged BCR microclusters.

### WASp Is Required for the Formation and Stabilization of Actin Foci Supporting Centralized Membrane Protrusions

**FIGURE 4:**
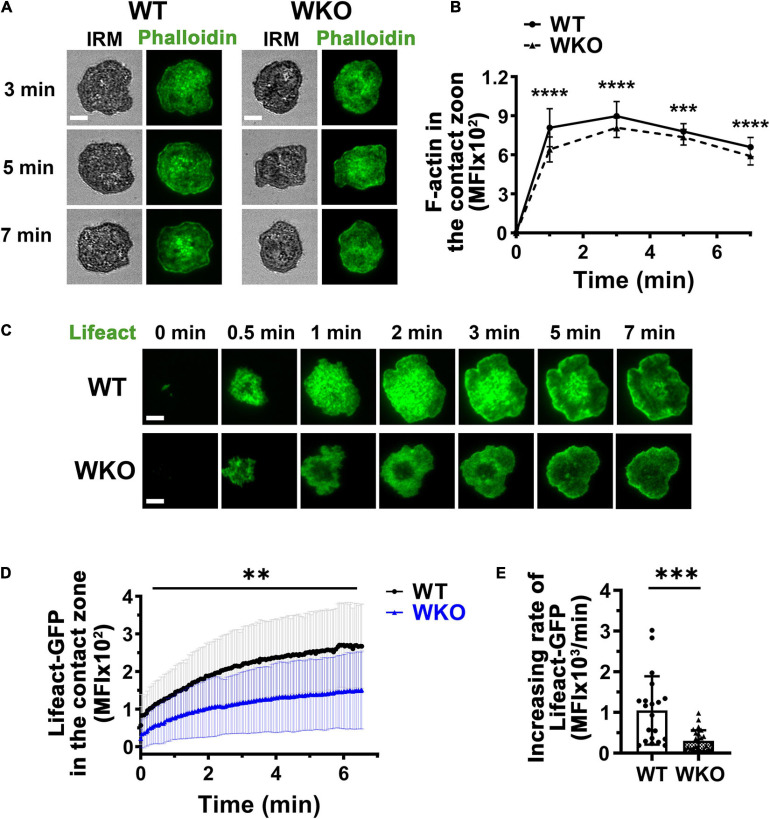
WASp is involved in F-actin accumulation in the GCB contact zone. **(A,B)** GCBs isolated from SRBC-immunized WT and WKO mice were incubated with Fab’-PLB, fixed, permeabilized, stained for F-actin with phalloidin, and imaged using IRM and TIRF. Shown are representative images **(A)** and the MFI of phalloidin in the GCB contact zone at different times **(B)**. **(C–E)** GCBs isolated from SRBC-immunized WT and WKO mice expressing Lifeact-GFP were incubated with Fab’-PLB and imaged live using TIRF. Shown are representative time-lapse TIRF images of Lifeact-GFP **(C)**, the MFI of Lifeact-GFP in the contact zone over time **(D)**, and the increasing rates of Lifeact-GFP MFI, measured as illustrated in Figure 2E. Data points represent individual cells. Scale bars, 3 μm. The results were the average (± SD) of 22∼50 individual cells per condition. *n* = 4. **p < 0.01, ***p < 0.001, and ****p < 0.0001, unpaired Student’s *t*-test or one-way ANOVA.

WASp is an actin nucleation-promoting factor that activates Arp2/3-mediated polymerization of branched actin (Padrick and Rosen, 2010). We evaluated the contribution of WASp to actin reorganization during GCB response to antigen-presenting surfaces using phalloidin staining and GCBs from Lifeact-GFP-expressing mice. Both phalloidin and Lifeact-GFP bind to F-actin, but phalloidin staining requires cell fixation and membrane permeabilization (Riedl et al., 2008; Melak et al., 2017). Analysis of TIRF images showed that the MFI of phalloidin staining in the contact zone of GCBs fixed at different times increased rapidly upon interacting with Fab’-PLB and peaked before 1 min (Figures 4A,B). The MFI of Lifeact-GFP in the contact zone of GCBs imaged live rose relatively slowly (Figures 4C,D and Supplementary Video 3). However, the MFI of both phalloidin and Lifeact-GFP in the contact zone of WKO GCBs were significantly reduced, compared to those of WT GCBs (Figures 4A–D and Supplementary Video 3). Furthermore, the initial rate of increase of Lifeact-GFP in the WKO GCB contact zone was significantly slower than that in the WT GCB contact zone (Figure 4E). Thus, WASp is primarily involved in the rapid accumulation of F-actin in the GCB contact zone.

**FIGURE 5:**
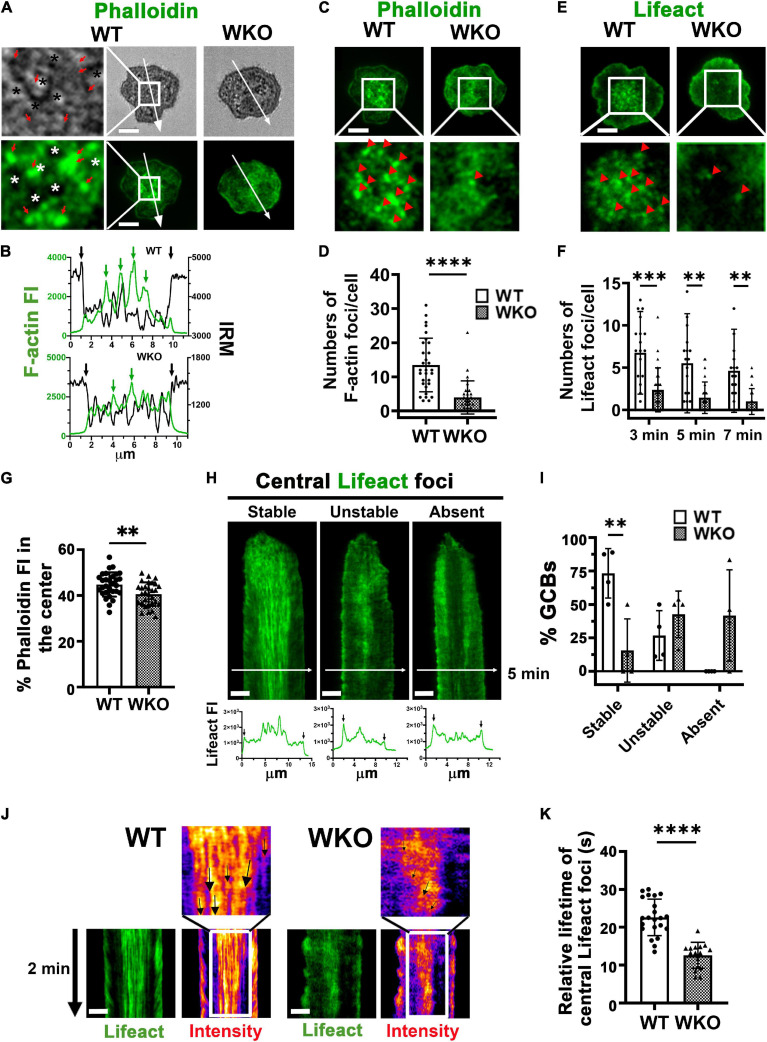
WASp is required for generating stable F-actin foci at the centralized membrane protrusions in the GCB contact zone. GCBs isolated from SRBC-immunized WT and WKO mice expressing or not Lifeact-GFP were incubated with Fab’-PLB and either fixed, permeabilized, stained with phalloidin and imaged, or imaged live using IRM and TIRF. **(A)** Representative IRM and TIRF images of phalloidin staining of WT and WKO GCBs at 7 min and enlarged images of the central area in the WT GCB contact zone (left panels). Star *, the plasma membrane areas pulled away from Fab’-PLB. Red arrows, the plasma membrane areas were in close contact with Fab’-PLB. **(B)** The FI of phalloidin staining and the density of IRM image along the arrowed lines of WT (top) and WKO (bottom) cells in **(A)**. **(C)** Representative images of fixed GCBs stained with phalloidin and their central area of the contact zone at 7 min. Red arrowheads, phalloidin stained F-actin foci identified by the MFI of individual foci that were 2 folds higher than the MFI of the entire contact zone. **(D)** The number of phalloidin-stained F-actin foci per GCB contact zone at 7 min. Data points represent individual cells. **(E)** Representative images of Lifeact-GFP expressing GCBs and their enlarged central area of the contact zone at 7 min. Red arrowheads, Lifeact-GFP F-actin foci identified by the MFI of individual foci that were 1.5 fold higher than the MFI of the entire contact zone. **(F)** The number of Lifeact-GFP foci per GCB contact zone at 3, 5, and 7 min. Data points represent individual cells. **(G)** Percentages of the phalloidin FI in the central area relative to the phalloidin TFI in the contact zone. Data points represent individual cells. **(H)** Representative kymographs generated from time-lapse images of Lifeact-GFP GCBs by TIRF and Lifeact-GFP FI line profiles at 5 min (white arrow lines) to show three phenotypes: stable, unstable, and no central Lifeact foci. Black arrows in line profiles indicate the outer edges of the GCB contact zone. **(I)** Percentages of cells with stable, unstable, and no Lifeact foci in the center of the contact zone. Data points represent independent experiments. **(J)** Representative kymographs of Lifeact-GFP-expressing GCBs and their intensity heat maps from 4 to 6 min. Arrows, tracks of individual Lifeact-GFP actin foci. **(K)** The relative lifetime of Lifeact-GFP F-actin foci was measured by the duration of individual foci that could be tracked in a randomly selected kymograph. Data points represent individual cells, averages of 3 foci per cell. Scale bars, 3 μm. The results were the average (± SD) of 20∼30 individual cells per condition. *n* = 3∼4. **p < 0.01, ***p < 0.001, and ****p < 0.0001, unpaired Student’s *t*-test or one-way ANOVA.

Previous studies have shown that the GCB IS consists of actin-rich protrusions engaging antigen on presenting surfaces (Nowosad et al., 2016; Kwak et al., 2018). Consistent with these early findings, we found F-actin puncta, as observed by phalloidin staining, concentrated at the central area of the WT GCB contact zone (Figure 5A), where signaling puncta were also located (Figure 1). The overlay of phalloidin FI and IRM intensity line profiles showed the colocalization of F-actin foci with the darker regions of IRM images, where the GCB membrane was in close apposition to antigen-presenting surfaces (Figures 5A,B, green arrows). However, we did not detect such F-actin foci at the GCB membrane area surrounding the central protrusions even though the IRM intensity showed its interaction with Fab’-PLB (Figures 5A,B). We identified local regions of actin enrichment as F-actin foci close to Fab’-PLB if their phalloidin (Figure 5C) and Lifeact-GFP (Figure 5E) MFI was 2 and 1.5 folds higher than the MFI of phalloidin and Lifeact-GFP over the entire contact zone, respectively. We found a significantly higher number of such F-actin foci in the center of the WT than the WKO contact zone (Figures 5A–F). This reduced number of F-actin foci was associated with a decrease in the percentage of phalloidin staining in the center of the WKO GCB contact zone compared to that in the WT GCB contact zone (Figure 5G). We examined the formation of these centralized actin foci using kymographs generated from time-lapse images of GCBs expressing Lifeact-GFP (Figure 5H). We found that ∼70% of WT GCBs formed F-actin foci during the first minute of interaction with antigen-presenting surfaces. These F-actin foci gradually became centralized and were detected up to 20 min (Stable) (Figure 5H, left panels, and Figure 5I). In the remainder of WT GCBs, F-actin foci formed but did not persist for very long (Unstable) (Figure 5H, middle panels, and Figure 5I). In contrast, only ∼14% of WKO GCBs formed stable central F-actin foci (Figure 5I). We did not detect any centralized F-actin foci in ∼43% WKO GCBs (Figure 5H, right panels, and Figure 5I). The remainder of the WKO GCBs exhibited unstable F-actin foci (Figure 5H, middle panels, and Figure 5I). We evaluated the relative lifetime of F-actin foci by measuring the duration of individual F-actin foci that could be detected in randomly selected GCB kymographs and their intensity heat maps (Figure 5J, arrows). The relative lifetime of F-actin foci in WKO GCBs was much shorter than those in WT GCBs (Figures 5J,K). These data indicate that WASp is essential for the generation and stabilization of centralized F-actin foci supporting the GCB membrane protrusions that engage antigen on presenting surfaces.

### WASp Contributes to the Actin Cytoskeleton Supporting the Lamellipodia and the Adherent Region Surrounding the Central Protrusions

**FIGURE 6:**
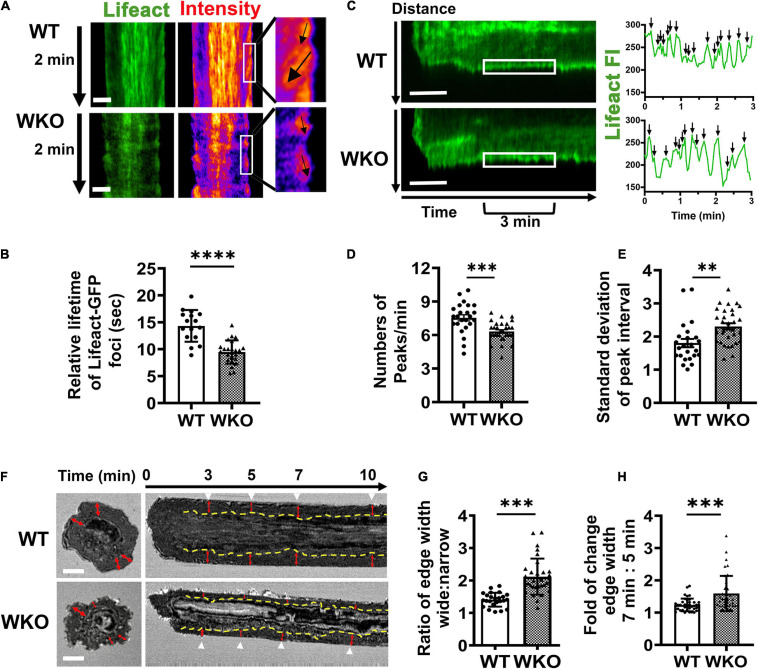
WASp is required for the formation of a steady adherent region surrounding the central signalosome. GCBs isolated from SRBC-immunized Lifeact-GFP mice were incubated with Fab’-PLB and imaged live using IRM and TIRF. **(A)** Representative kymographs from 2.5 to 4.5 min and their intensity heat maps generated from Lifeact-GFP time-lapse images. White box, peripheral regions for 1 min. Black arrows, tracks of Lifeact foci at an edge region. **(B)** Relative lifetimes of Lifeact-GFP foci were evaluated by the duration of Lifeact-GFP foci detected in randomly selected kymographs. Data points represent individual cells, the average of 8∼10 Lifeact foci tracks per cell. **(C)** Representative Lifeact-GFP kymographs (from the center to the periphery of the contact zone). White box, peripheral regions between 3 and 6 min. FI in the peripheral region (white box) over time was shown in the right panels. Black arrows, individual Lifeact-GFP FI peaks. **(D)** Numbers of Lifeact-GFP FI peaks per min between 3 and 6 min. **(E)** Standard deviation (SD) of time intervals between peaks. **(F)** Representative IRM images at 7 min and kymographs generated from time-lapse IRM images of WT and WKO GCBs. Yellow dashed lines indicate the border between the periphery and center regions of the contact zone based on IRM density. Red arrows indicate the width of the periphery area. White arrow, time. **(G)** The ratio of the periphery width at the wider side relative to the narrow side. Data points represent the average of the ratios at 3, 5, 7, and 10 min in individual cells. **(H)** The fold of change in the periphery width between 5 and 7 min. Data points represent individual cells. Scale bars, 3 μm. The results were the average (± SD) of 20∼30 individual cells per condition. *n* = 4. **p < 0.01, ***p < 0.001, and ****p < 0.0001, unpaired Student’s *t*-test or one-way ANOVA.

In addition to the centralized F-actin foci, we observed actin structures at the outer edge of the contact zone, supporting the spreading membrane (Figure 6A and Supplementary Video 3). We determined their relative lifetime using kymographs generated from time-lapse images of Lifeact-GFP-expressing GCBs (Figure 6A) as described for the relative lifetime of central F-actin foci. Similar to the central F-actin foci, the actin structures at the outer edge of the WKO GCB contact zone had significantly shorter lifetimes than those of WT GCBs (Figure 6B). We further measured the number of Lifeact-GFP FI peaks per minute (Figures 6C,D) and the standard deviation of the time between two Lifeact-GFP peaks (Figures 6C,E) using kymographs to reflect the frequency and the regularity of this actin structure at the leading edge of the spreading membrane. We found that the Lifeact-GFP FI peaks appeared at the outer edge of the WKO GCB contact zone much less frequently and with less regularity than those in WT GCBs (Figures 6C–E). To examine the impact of such changes in the actin cytoskeleton caused by WKO on the interaction of GCBs with antigen-presenting surfaces, we analyzed time-lapse IRM images using kymographs (Figure 6F). In the contact zone of WT GCBs, we observed a ∼2 μm wide dark region (indicating adhesion of the GCB plasma membrane to antigen-presenting surfaces) surrounding the centralized protrusions after reaching maximal spread area (Figure 6F, top panels, red arrows). The width of this adherent region in WKO GCBs appeared to be more variable over time and more asymmetrical than in WT GCBs (Figure 6F, bottom panels, red arrows). To examine this adherent region closely, we determined the width ratio of the adherent region at two opposite sides in individual contact zones at 3, 5, 7, and 10 min (Figure 6G) and the fold change in the width of the adherent region between 5 and 7 min (Figure 6H). WKO GBCs displayed significantly higher ratios of the adherent region width between the two sides of individual contact zones (Figure 6G) and significantly greater fold changes between 5 and 7 min (Figure 6H) than WT GCBs. These results suggest that WKO destabilizes this adherent region. Thus, WASp contributes to the actin cytoskeletal network that maintains the adhesion of GCBs to antigen-presenting surfaces, thereby stabilizing the IS.

## Discussion

Germinal centers are critical for selecting high-affinity antigen-specific B-cells and eliminating non-specific B-cells generated during somatic hypermutation (Victora and Nussenzweig, 2012; Mesin et al., 2016). A lack of antibody responses to vaccinations and infections and an accumulation of autoantibodies in WAS patients and mouse models suggest a failure of GCBs in the affinity maturation process (Notarangelo et al., 2008; Bosticardo et al., 2009; Becker-Herman et al., 2011). This study has revealed that WASp is essential for building the unique architecture of the GCB IS. WASp is responsible for generating and stabilizing actin structures that support and maintain membrane protrusions in the center of the GCB IS. WASp also contributes to the actin structures that drive GCB spreading and adhesion to antigen-presenting surfaces, stabilizing the GCB IS. GCBs rely on this unique architecture of the IS to discriminate the affinity of antigen presented on the surface of antigen-presenting cells, activate BCR signaling, and capture antigen for processing and presentation (Nowosad et al., 2016; Kwak et al., 2018).

Recent studies have shown that GCBs from human tonsils and mouse spleens engage membrane antigen through actin-rich pod-like membrane protrusions and form BCR microclusters at the tips of these protrusions (Nowosad et al., 2016; Kwak et al., 2018). This organization is different from the naïve B-cell IS. The naïve B-cell plasma membrane interacting with antigen-presenting surfaces is relatively smooth, allowing BCR microclusters to merge into a central cluster. In contrast, the pod-like membrane protrusions prohibit BCR microclusters from growing and merging. This unique GCB IS architecture has been shown to provide GCBs an enhanced ability to sense their binding affinity to antigen, as the stability of pod-like membrane protrusions increases with antigen affinity (Nowosad et al., 2016; Kwak et al., 2018). Using high-resolution IRM and TIRF imaging, this study extends these findings by showing that these membrane protrusions concentrate in the center of the GCB contact zone and are relatively stable when GCBs interact with PLB coated with a relatively high density of Fab’ fragment of anti-IgM + G antibody. As PLBs provide high-avidity binding to surface BCRs, the centralized, stable protrusions probably reflect the IS characteristics in GCBs interacting with high-affinity antigen. How antigen affinity and the stiffness of antigen-presenting surfaces influence the GCB IS architecture remains an interesting question.

This study further shows that activated signaling molecules, including both positive (CD79, Syk, and Akt) and negative (SHP1) signaling molecules, appear at the protrusions at similar times. The peak levels of phosphorylated CD79a and Syk persist at least for 20 min. In contrast, stimulatory kinases in naïve B-cells interacting with the same type of Fab’-PLB are first activated and then down-regulated by activated phosphatases (Liu et al., 2011, 2013a; Seeley-Fallen et al., 2014). Our findings are consistent with the previous observation that the activity of phosphatases is enhanced in GCBs (Khalil et al., 2012) and increases at similar times as stimulatory kinases in response to antigenic stimulation (Kwak et al., 2018). Together, these data suggest that the phosphatases SHP1 and SHIP1 work with stimulatory kinases to regulate the level of persistent BCR signaling in germinal center B-cells rather than deactivate BCR signaling in naïve B-cells. We also noticed that stimulatory kinases and inhibitory phosphatases distribute differentially at the membrane protrusions, even though they are activated in GCBs simultaneously. Activated CD79, Syk, and SHP1 fill the contact region of the membrane protrusions and the outer edge of the contact zone, while activated Akt and SHIP1 appear as puncta, sparsely decorating the membrane protrusions and the outer edge of the contact zone, respectively. These observations suggest that GCBs organize signaling molecules into distinct microclusters and maintain them for persistent signaling through pod-like membrane protrusions. However, our studies using TIRF were limited to the GCB contact membrane. High-resolution three-dimensional imaging is required for fully visualizing and characterizing the organization of GCB signalosomes.

WASp has been shown to have B-cell intrinsic roles in the germinal center, causing GCB hyper-proliferation and plasma cell differentiation in WASp-deficient mouse models (Becker-Herman et al., 2011). However, the cellular mechanism by which WASp regulates GCB affinity maturation remains elusive. WASp activates the actin nucleation factor Arp2/3, downstream of Cdc42 and phosphatidylinositol-4,5-biphosphates (Bi and Zigmond, 1999; Padrick and Rosen, 2010). Activated Arp2/3 polymerizes branched actin filaments, which are known to drive lamellipodia of migrating and spreading cells (Bisi et al., 2013; Alekhina et al., 2017). Therefore, it is not surprising to find that WASp contributes significantly to GCB spreading on antigen-presenting surfaces, particularly the spreading kinetics. WASp plays a similar role in naïve B-cells (Liu et al., 2013a). Spreading expands B-cell contact with antigen-presenting surfaces, amplifying BCR clustering and signaling (Fleire et al., 2006; Harwood and Batista, 2010).

In addition to its role in GCB spreading, the new finding of this study is that WASp is essential for the formation and stabilization of pod-like membrane protrusions, the unique membrane structures of the GCB IS. WASp is responsible for the generation and/or extended lifetime of actin foci that support the membrane protrusions at the center of the GCB contact zone, as both the number of actin foci and their relative lifetime are reduced in WKO GCBs. This finding suggests that actin structures supporting pod-like membrane protrusions are based on branched actin networks. Furthermore, WASp is also required for the stability and regularity of branched actin structures at the outer edge of the GCB contact membrane after rapid spreading. These actin structures, which appear to be tread milling, function to maintain the GCB contact area and the GCB adherence to antigen-presenting surfaces surrounding the centralized membrane protrusions. The role of these actin structures in GCBs is opposite to what was observed in naïve B-cells, where actin at the outer edge of the contact zone drives B-cell contraction following spreading, which promotes signaling attenuation (Liu et al., 2013a; Seeley-Fallen et al., 2014). This functional difference in the actin cytoskeleton between GCBs and naïve B-cells likely contributes to their distinct BCR signaling signatures and a heightened ability of GCBs to distinguish antigen-binding affinity compared to naïve B-cells (Khalil et al., 2012; Nowosad et al., 2016; Kwak et al., 2018; Luo et al., 2019). The role of WASp in the generation and maintenance of actin structures supporting the unique architecture of the GCB IS may explain the defective germinal center reaction in WAS patients and mouse models. WASp-deficiency causes GCBs to generates less and/or relatively unstable membrane protrusions, which likely reduces the ability of GCBs to distinguish antigen-binding affinity, causing failures in selecting high-affinity antigen-specific B-cells.

The reductions in BCR phosphorylation, BCR clustering, and cell spreading in WKO GCBs compared to WT GCBs suggest that WASp promotes BCR activation by increasing cell spreading and BCR clustering at the contact zone. These findings are similar to what we observed in WKO native B-cells (Liu et al., 2013a), suggesting a common role for WASp in naïve B-cells and GCBs, besides its GCB-specific functions. Using single molecule imaging, we have recently shown that the mobility of surface BCRs is significantly lower in WKO native B-cells than in WT naïve B cells (Rey-Suarez et al., 2020), implying that WASp promotes BCR clustering by mobilizing BCRs on the B-cell surface. Interestingly, along with the reduced BCR clustering and activation in WKO GCBs, the levels of two major phosphatases in the contact zone are differentially altered, with pSHIP1 increasing and pSHP1 decreasing, suggesting that WASp differentially regulates these two phosphatases. The implication of such opposing alterations of SHIP1 and SHP1 activation in WKO GCBs is unknown. We have also noticed that the impact of WASp-deficiency on the organization of micro-signaling clusters is much more than its effect on the level of BCR proximal signaling. This is likely the result of the saturated strength of activation by a relatively high-density of Fab’ on PLB, overcoming signaling defects caused by WASp deficiency. Future studies with graded antigen density and affinity will further reveal the mechanism by which WASp regulates GCB affinity maturation.

The moderate reduction in cell spreading, BCR clustering, and signaling in WKO GCBs indicates that WASp is not essential for BCR signaling in GCBs. The WASp family has additional members, including N-WASp and WAVE, which can all activate Arp2/3-mediated branched actin polymerization (Padrick and Rosen, 2010; Oda and Eto, 2013; Alekhina et al., 2017). We have previously shown that both WASp and N-WASp are required for cell spreading, BCR clustering, and signaling in naïve B-cells. Naïve B-cells from WASp and N-WASp (B-cell-specific) double knockout mice fail to spread, cluster surface BCRs, and activate BCRs when interacting with the same Fab’-PLB (Liu et al., 2013a). These results suggest that N-WASp can functionally compensate for the lack of WASp in GCBs but cannot exclude WAVE’s possible roles in GCBs. How these actin nucleation promoting factors work together in GCBs to facilitate affinity maturation is a subject of our future interest.

In summary, this study has identified a role for WASp and WASp-activated branched actin polymerization in the generation and maintenance of the unique architecture of the GCB IS. This unique architecture is essential for GCBs to discriminate antigen-binding affinity for the selection of high-affinity antigen-specific B-cells to mount antibody responses. The defective GCB IS architecture provides a cellular mechanism by which WASp deficiency causes immune disorders.

## Data Availability Statement

The original contributions presented in the study are included in the article/Supplementary Material, further inquiries can be directed to the corresponding author/s.

## Ethics Statement

The animal study was reviewed and approved by the Institutional Animal Care and Use Committee (IACUC) at the University of Maryland (College Park, MD, United States) and Chongqing Medical University (Chongqing, China).

## Author Contributions

YL, WS, AU, and XZ established the study design and provided the research strategy. YL and AB performed the experiments and the data analysis. YL and WS prepared and wrote the manuscript. All authors read and approved the manuscript.

## Conflict of Interest

The authors declare that the research was conducted in the absence of any commercial or financial relationships that could be construed as a potential conflict of interest.
