# Corrigendum: WASp Is Crucial for the Unique Architecture of the Immunological Synapse in Germinal Center B-Cells

**DOI:** 10.3389/fcell.2022.819050

**Published:** 2022-02-09

**Authors:** Yanan Li, Anshuman Bhanja, Arpita Upadhyaya, Xiaodong Zhao, Wenxia Song

**Affiliations:** ^1^ Department of Rheumatology and Immunology, Children’s Hospital of Chongqing Medical University, Chongqing, China; ^2^ Ministry of Education Key Laboratory of Child Development and Disorders, Chongqing Key Laboratory of Child Infection and Immunity, China International Science and Technology Cooperation Base of Child Development and Critical Disorders, National Clinical Research Center for Child Health and Disorders, Chongqing, China; ^3^ Department of Cell Biology and Molecular Genetics, University of Maryland, College Park, College Park, MD, United States; ^4^ Department of Physics, University of Maryland, College Park, College Park, MD, United States; ^5^ Institute for Physical Science and Technology, University of Maryland, College Park, College Park, MD, United States

**Keywords:** B-lymphocytes, germinal center, actin, WASp, signal transduction, immunological synapse

There is an error in the **Funding** statement. The correct funding number for “XZ” is “81620108014.”

The authors apologize for this error and state that this does not change the scientific conclusions of the article in any way. The original article has been updated.

