# Supplementary figures and images for: WASp Is Crucial for the Unique Architecture of the Immunological Synapse in Germinal Center B-Cells
Source: Front Cell Dev Biol. 2021 Jun 14;9:646077. doi: 10.3389/fcell.2021.646077 (PMC8236648; doi:10.3389/fcell.2021.646077)

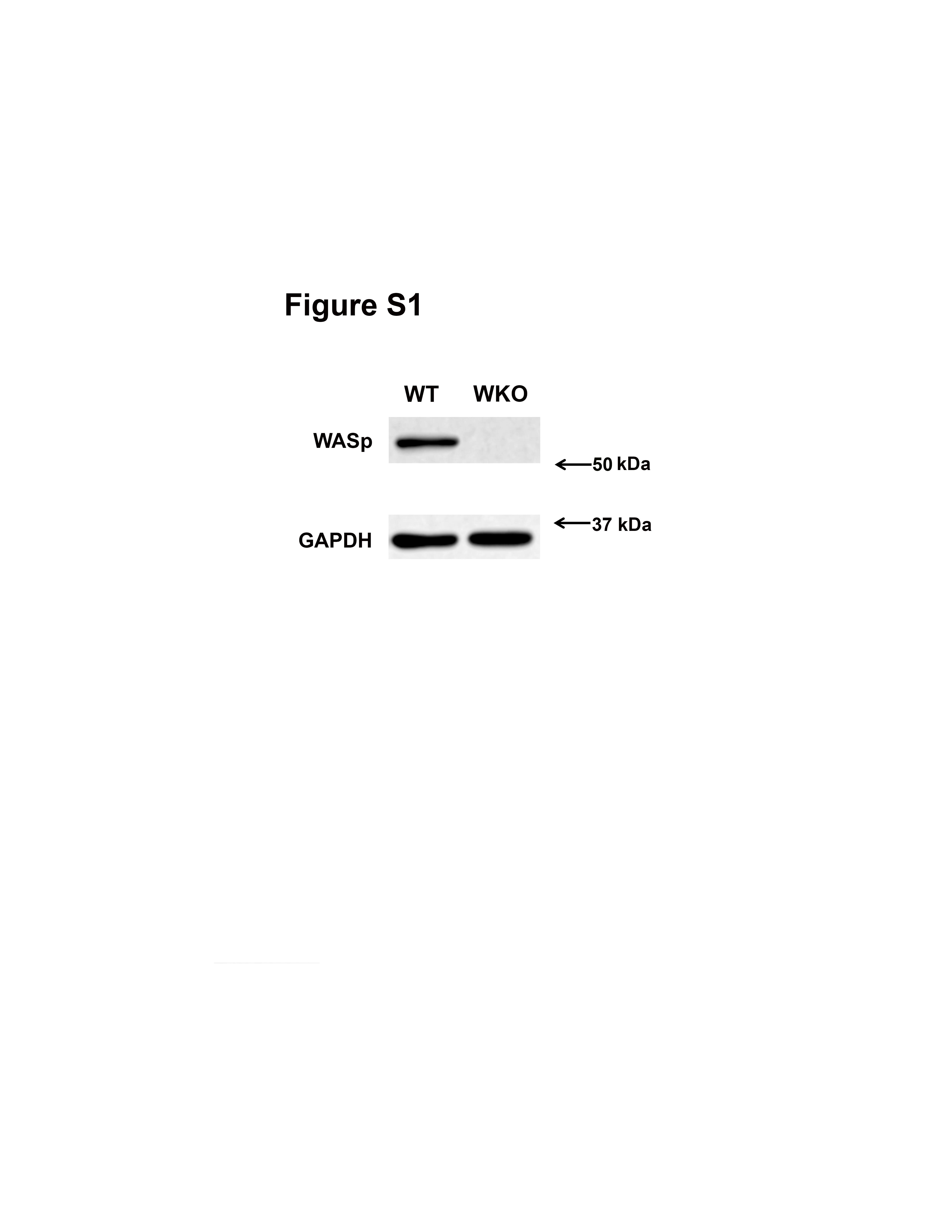

Supplement: Supplementary file 1 [file Image_1.tif]

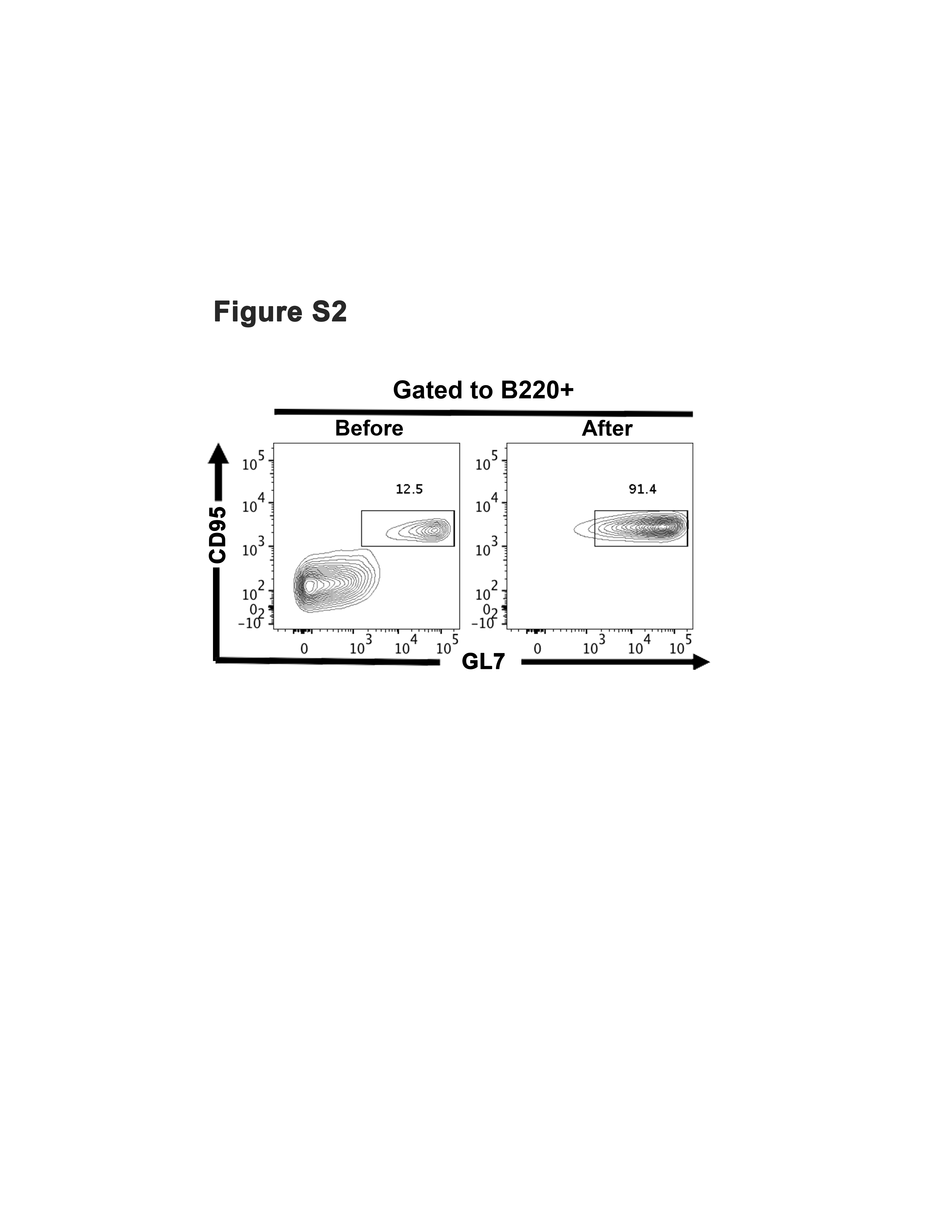

Supplement: Supplementary file 2 [file Image_2.TIF]

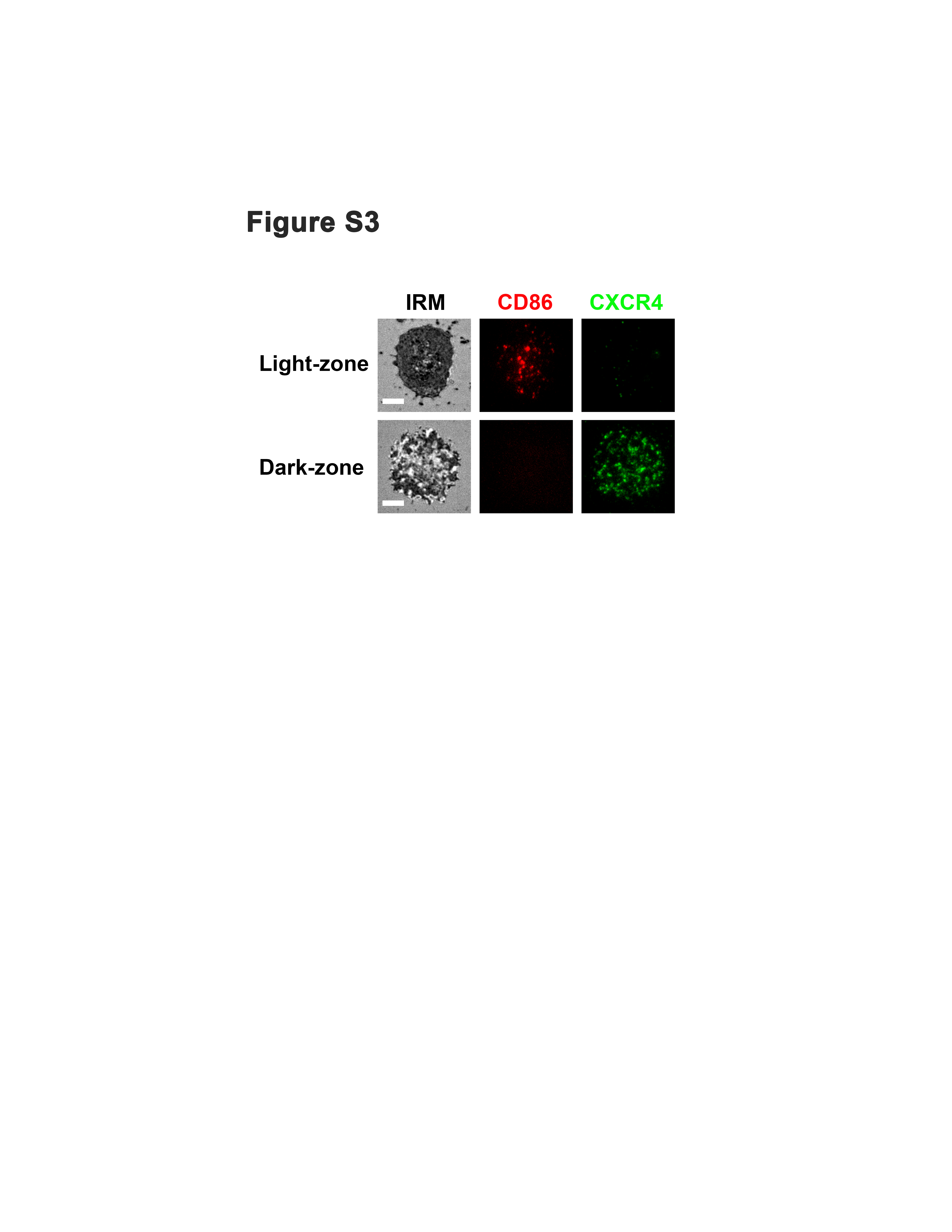

Supplement: Supplementary file 3 [file Image_3.TIF]

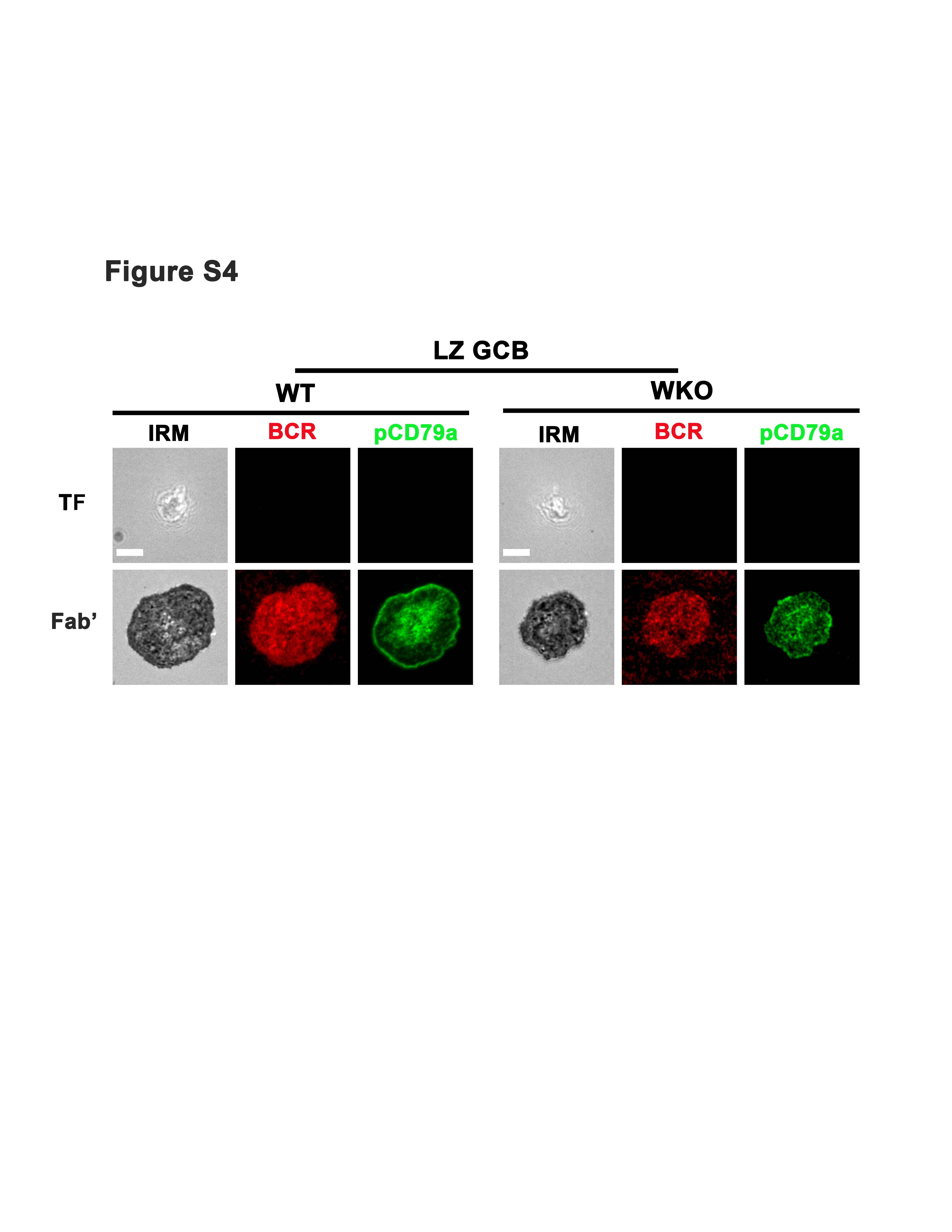

Supplement: Supplementary file 4 [file Image_4.TIF]

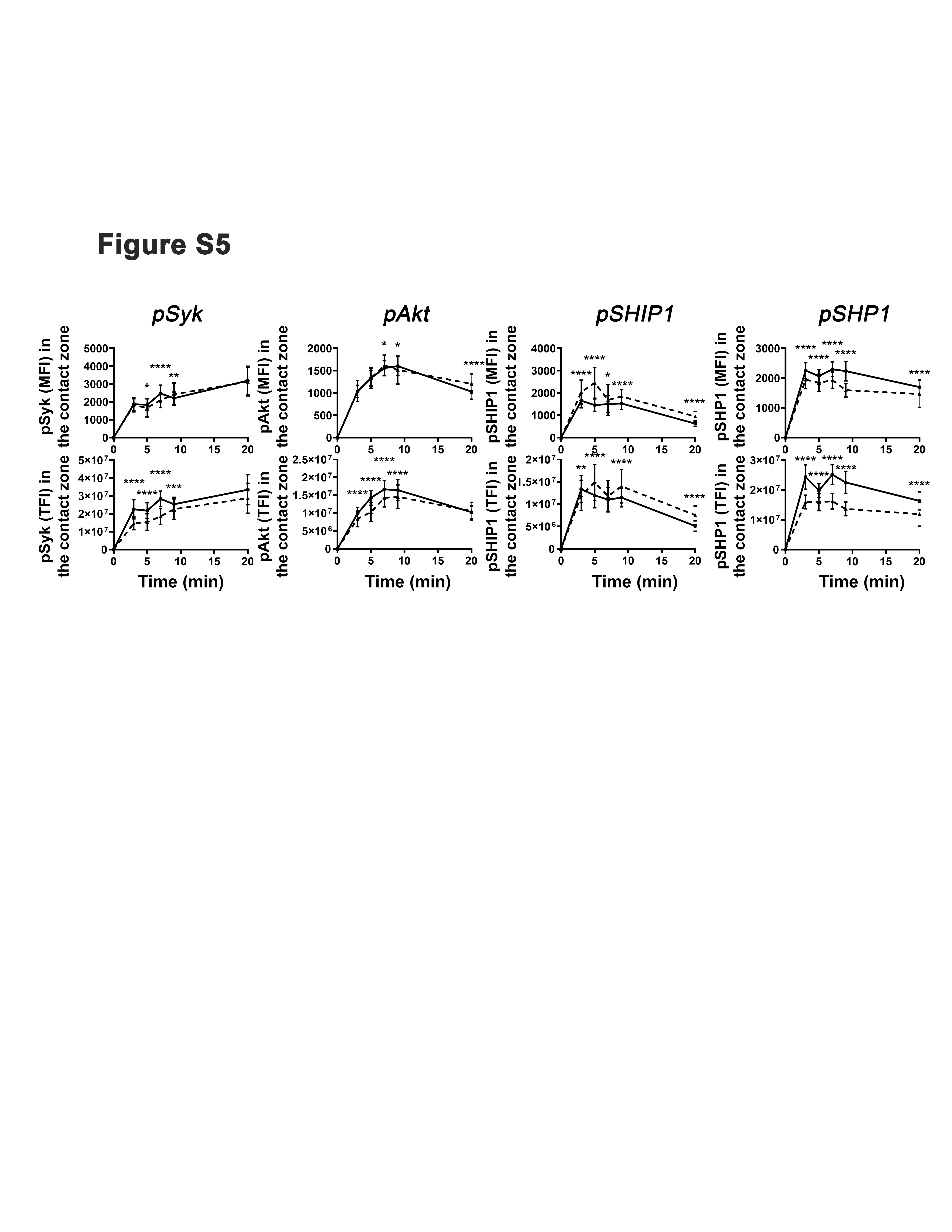

Supplement: Supplementary file 5 [file Image_5.TIF]

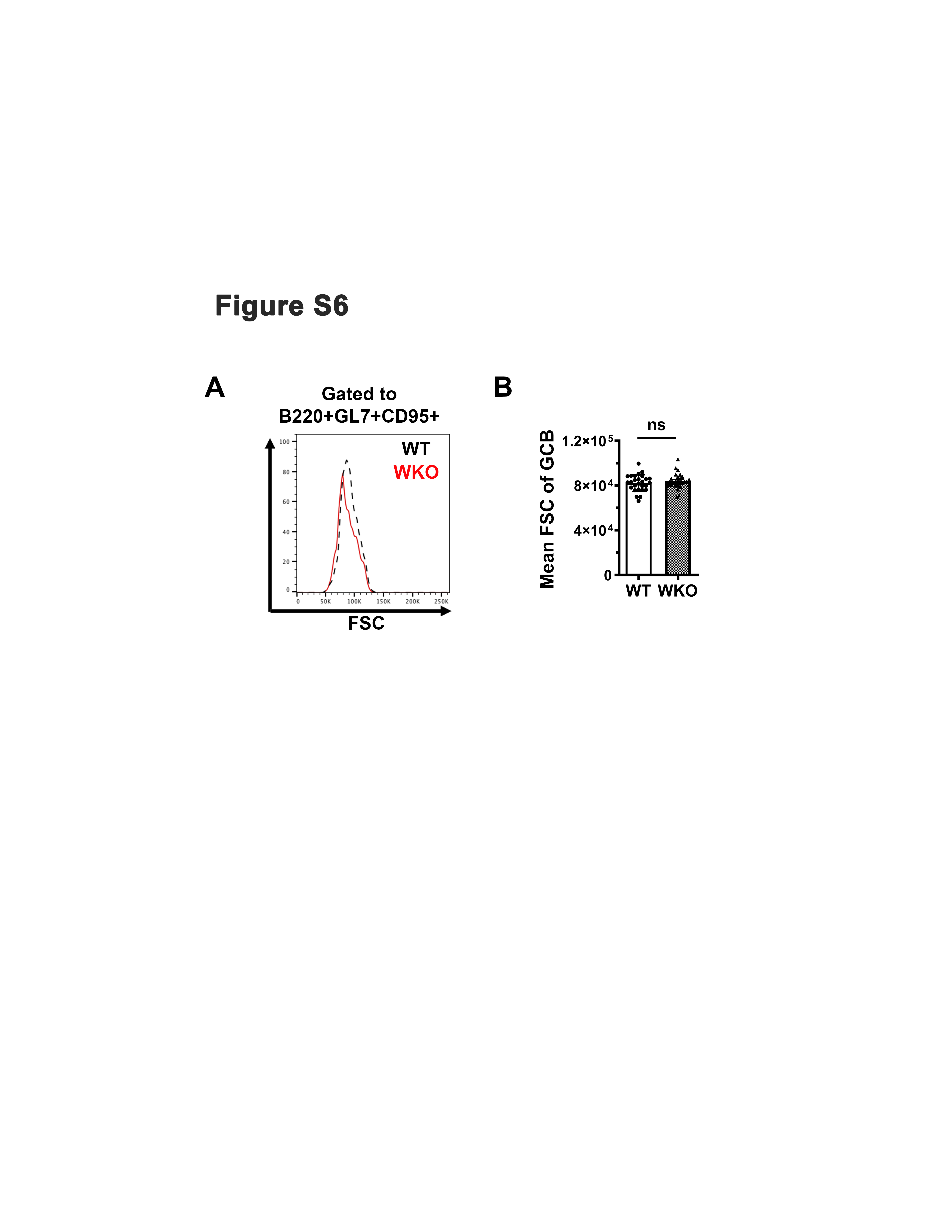

Supplement: Supplementary file 6 [file Image_6.TIF]

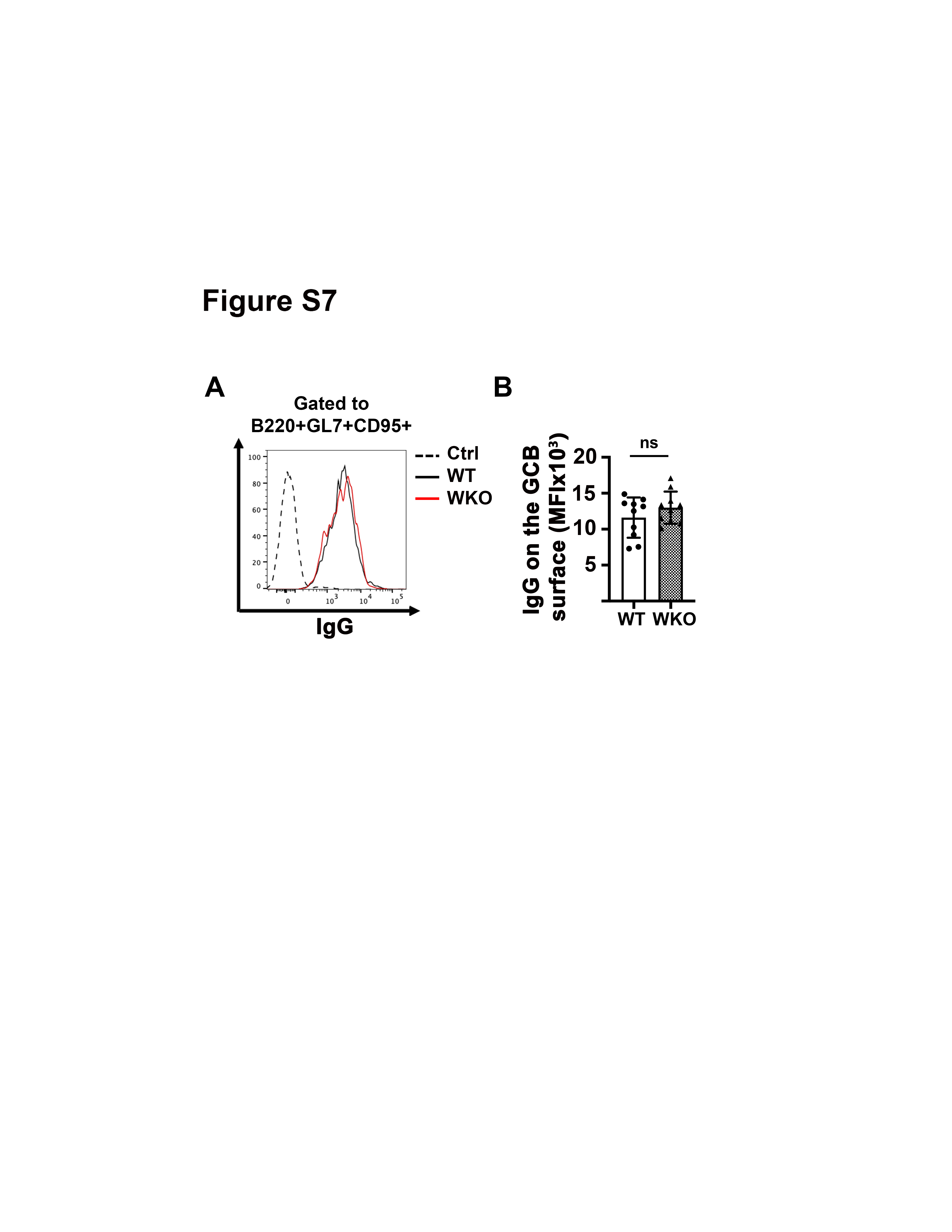

Supplement: Supplementary file 7 [file Image_7.TIF]
