# Supplementary material for: WASp Is Crucial for the Unique Architecture of the Immunological Synapse in Germinal Center B-Cells
Source: Front Cell Dev Biol. 2021 Jun 14;9:646077. doi: 10.3389/fcell.2021.646077 (PMC8236648; doi:10.3389/fcell.2021.646077)
Supplement: Supplementary file 11 [file Data_Sheet_1.docx]

**SUPPLEMENTARY MATERIALS**

**Supplementary Figure S1. Splenocytes from WKO mice lack WASp expression.** Splenocytes from WT and WKO mice were lysed, and cell lysates were analyzed by western blotting, probing for WASp. The blots were stripped and probed for GAPDH as loading controls. Shown are representative blots. n=4.

**Supplementary Figure S2. Isolation of germinal center B-cells (GCBs).** GCBs were isolated from sheep red blood cell (SRBC) immunized mice using antibody-coated magnetic beads. Splenic mononuclear cells and isolated GCBs were stained for the B-cell marker B220 and the GCB markers, GL7 and CD95, and analyzed using flow cytometry. Shown are representative dot plots.

**Supplementary Figure S3. Identification of light zone and dark zone GCBs.** GCBs isolated from SRBC-immunized mice were stained for CD86 and CXCR4 before incubated with Fab’-PLB and analyzed using epifluorescence microscope, TIRF, and IRM. Shown are representative IRM and TIRF images at 5 min. Light zone GCBs were identified by relatively high CD86 staining, relatively low CXCR4 staining, and relatively darker IRM images. Scale bar, 3 μm.

**Supplementary Figure S4. GCBs interacting with transferrin (Tf)-PLB as negative controls.** GCBs from SRPC-immunized mice were incubated with Tf- or Fab’-coated PLB at 37^o^C for 7 min. Cells were then fixed, permeabilized, stained for pCD79a, pSyk, pAkt, pShip1, and pSHP1, and imaged by IRM and TIRF. Shown are representative IRM and TIRF images of GCBs stained for pCD79a. GCBs interacting with Tf-PLB and stained for other signaling molecules were similar to those stained for pCD79a. Scale bar, 3 μm.

**Supplementary Figure S5. BCR signaling in the contact zone of WKO GCBs is altered compared to WT GCBs.** GCBs from SRBC-immunized mice were incubated with Fab’-PLB, fixed at different times, permeabilized, stained for pSyk, pAkt, pSHIP1, or pSHP1, and imaged by IRM and TIRF. The MFI (top plots) and the TFI (bottom plots) of pSyk, pAkt, pSHIP1, and pSHP1 in the contact zone of WT and WKO GCBs was measured using IRM and TIRF images and NIH ImageJ software. The results were the average (±SD) of >80 individual cells per condition. n=3~4. **p*<0.05, ***p*<0.01, ****p*<0.001, *****p*<0.0001, unpaired Student’s *t*-test or one-way ANOVA.

**Supplementary Figure S6. WT and WKO GCBs have similar sizes.** Splenocytes from SRBC-immunized WT and WKO mice were stained for B220, GL7, and CD95 and analyzed using flow cytometry. B220^+^GL7^+^CD95^+^ cells were gated. **(A)** Representative histograms of forward scatter (FSC) of WT and WKO GCBs. **(B)** The average FSC (±SD) of GCBs. n=25.

**Supplementary Figure S7. WKO does not reduce the surface levels of the BCR.** Splenocytes from immunized WT and WKO mice were stained with antibodies specific for B220, GL7, CD95 and IgG, and analyzed by a BD FACS Canto II flow cytometer and Flowjo software. B220^+^GL7^+^CD95^+^ cells were gated. **(A)** Representative histograms of surface IgG MFI of WT and WKO GCBs. **(B)** The average MFI (±SD) of IgG on GCBs. n=7.

**Supplementary Video 1. GCB spreading on Fab’-PLB by IRM.** GCBs isolated from SRBC-immunized WT and WKO mice were incubated with Fab’-PLB at 37^o^C and 5% CO_2_. IRM images were acquired a frame every 2 sec. Shown are videos played at 20 frames per sec.

**Supplementary Video 2. BCR clustering in the GCB contact zone.** GCBs isolated from SRBC-immunized WT and WKO mice were incubated with AF546-Fab’-PLB at 37^o^C and 5% CO_2_. TIRF images of AF546 were acquired a frame every 2 sec. Shown are videos played at 20 frames per sec.

**Supplementary Video 3. The reorganization of the actin cytoskeleton in the GCB contact zone.** GCBs isolated from SRBC-immunized WT and WKO mice expressing Lifeact-GFP were incubated with Fab’-PLB at 37^o^C and 5% CO_2_. TIRF images of Lifeact-GFP were acquired a frame every 2 sec. Shown are videos played at 20 frames per sec.
